# New Insights into Solid Form Stability and Hydrate Formation: *o*-Phenanthroline HCl and Neocuproine HCl

**DOI:** 10.3390/molecules22122238

**Published:** 2017-12-15

**Authors:** Doris E. Braun, Katharina Raabe, Anna Schneeberger, Volker Kahlenberg, Ulrich J. Griesser

**Affiliations:** 1 Institute of Pharmacy, University of Innsbruck, Innrain 52c, 6020 Innsbruck, Austria; katharina.raabe@student.uibk.ac.at (K.R.); anna.schneeberger@student.uibk.ac.at (A.S.); ulrich.griesser@uibk.ac.at (U.J.G.); 2 Institute of Mineralogy and Petrography, University of Innsbruck, Innrain 52, 6020 Innsbruck, Austria; volker.kahlenberg@uibk.ac.at

**Keywords:** 1,10-phenanthroline hydrochloride, neocuproine hydrochloride, hydrate, stability, thermal analysis, moisture sorption/desorption analysis, X-ray diffraction, intermolecular energies, crystal structure prediction

## Abstract

The moisture- and temperature dependent stabilities and interrelation pathways of the practically relevant solid forms of *o*-phenanthroline HCl (**1**) and neocuproine HCl (**2**) were investigated using thermal analytical techniques (HSM, DSC and TGA) and gravimetric moisture sorption/desorption studies. The experimental stability data were correlated with the structural changes observed upon dehydration and the pairwise interaction and lattice energies calculated. For **1** the monohydrate was identified as the only stable form under conditions of RH typically found during production and storage, but at RH values >80% deliquescence occurs. The second compound, **2**, forms an anhydrate and two different hydrates, mono- (**2-Hy1**) and trihydrate (**2-Hy3**). The **2-Hy1** structure was solved from SCXRD data and the anhydrate structure derived from a combination of PXRD and CSP. Depending on the environmental conditions (moisture) either **2-Hy1** or **2-Hy3** is the most sable solid form of **2** at RT. The monohydrates **1-Hy1** and **2-Hy1** show a high enthalpic stabilization (≥20 kJ mol^−1^) relative to the anhydrates. The anhydrates are unstable at ambient conditions and readily transform to the monohydrates even in the presence of traces of moisture. This study demonstrates how the right combination of experiment and theory can unravel the properties and interconversion pathways of solid forms.

## 1. Introduction

Organic molecules often occur in several crystalline forms, such as polymorphs (same chemical composition), hydrates (water-adducts), and solvates (organic solvent-adducts). These different solid forms are important research targets, particularly in the pharmaceutical and other fine-chemical industry, because they commonly have different physical or chemical solid-state properties, such as solubility, dissolution rate, density, chemical and physical stability which may have considerable consequences for manufacturing processes and the performance of final products [1,2,3]. An important aspect in the characterization of solid forms is their stability and transformation behavior under different environmental conditions (temperature, humidity, and pressure), as they may occur during drug manifold manufacturing processes or during storage of the compound/product.

Depending on the water activity and the temperature a hydrate can be the most stable form or unstable, which must be considered in the selection of certain manufacturing processes such as granulation. Moreover, the dehydration and rehydration behavior of a compound can be fairly complex and may involve multiple forms (for example [4]), which requires a thorough investigation of such interchanges for both practical and regulatory reasons. However, in-depth investigations and a sound understanding of the reasons for hydrate formation is also very important to support the progresses in computational efforts aiming at reliable predictions of hydrate formation and stability. Based on the hydration/dehydration mechanisms, the corresponding continuity/discontinuity of the sorption/desorption behavior, and structural changes, hydrates are commonly grouped into two main classes, stoichiometric and non-stoichiometric hydrates. Stoichiometric hydrates have a well-defined water content and the crystal structure is clearly different to that of the dehydration product. If the water can fully or partly escape from a hydrate without significant changes of the crystal structure (except anisotropic distortions of the structure depending on the number of accommodated water molecules), then we speak of non-stoichiometric hydrates [5,6].

**Figure 1:**
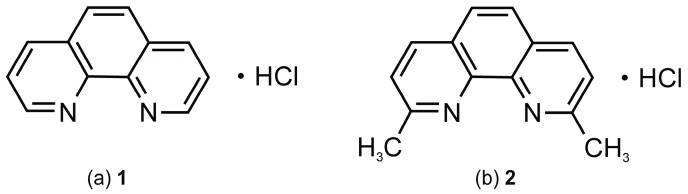
Molecular diagrams of (**a**) 1,10-phenanthroline hydrochloride (**1**) and (**b**) neocuproine HCl (**2**).

In this study we have chosen two HCl salts, 1,10-phenanthroline HCl (**1**) and 2,9-dimethyl-1,10-phenanthroline (neocuproine) HCl (**2**, Figure 1) as model compounds for ongoing experimental and computational studies aiming at a deeper understanding of the formation and behavior of hydrates of small organic molecules. Despite the fact that the free bases of these two compounds are heavily used, hardly any information about the solid state properties of these compounds can be found in the scientific literature [7]. The free base of **1** is used as a complexing agent for iron and other metal ions in chemical and biological systems. A classical use is that of the Fe(II)-complex (ferroin) as an indicator in redox titrations such as cerimetric titrations and as a diagnostic agent in biological applications because of its ability to block photosynthesis and metallopeptidases. The methylated derivative **2** is a chelating agent for copper(I) ions and is particularly used as a clinical reagent (blood glucose assay) and for spectrophotometric determination of copper [8]. For **1** the crystal structures of two distinct forms have been determined, an anhydrate **1-I** (CSD-Refcode [9]: CUZDIK [10]) and a monohydrate **1-Hy1** (PHOLCL/PHOLCL01 [11,12]). The two solid forms have different CAS numbers (3829-86-5 and 18851-33-7, for **1-I** and **1-Hy1**, respectively). Furthermore, **1** forms an ionic co-crystal [13] with *o*-phenanthroline (NIDXUT [14]) and its monohydrate structure is isostructural with the monohydrate of *o*-phenanthroline HBr (PHOLBR01 [15]). For the second salt, **2**, the only indication of different solid forms may be derived from the fact that distinct CAS numbers exist: water free (7296-20-0), monohydrate (303136-82-5) and an undefined hydrate stoichiometry (332360-00-6).

In the present study we seek to establish the reasons for hydrate formation and to unravel why for the two HCl salts the hydrates are the most stable solid-state forms at ambient conditions. The present work expands and complements our previous studies on stoichiometric hydrates (e.g.,: morphinanes [16]). A broad range of experimental techniques (HSM, DSC, TGA, PXRD, gravimetric moisture sorption/desorption analysis) and computational approaches (CSP, pairwise intermolecular energy calculations) were applied to resolve the structural, stability and interrelation pathways of neat and hydrate forms of **1** and **2**. Furthermore, the first structures for **2** have been solved and derived from SCXRD data and by combining CSP and PXRD, respectively.

## 2. Results

The experimental solid form screen for hydrates and neat forms encompassed RT solvent evaporation, sublimation and moisture sorption/desorption studies. The solvent evaporation screen from water, methanol and methanol/water mixtures covering *a*_w_ ranging from 0 to 1 in 0.1 steps resulted for **1** in all experiments in the known monohydrate (**1-Hy1**). Crystallization from a saturated (25 °C) chloroform solution, excluding moisture, lead to an unstable chloroform solvate. Due to its instability we were not able to investigate whether this phase corresponds to the tris(1,10-phenanthrolin-1-ium) dichloride (hydrogen chloride) chloride chloroform solvate described by Hensen et al. [10]. For **2**, depending on *a*_w_ of the used solvent, the evaporation experiments resulted either in the known monohydrate (**2-Hy1**) or if *a*_w_ was approx. 0.4 or higher in a trihydrate (**2-Hy3**). Neither for **1** nor **2** did solvent evaporation experiments lead to an anhydrous form. The latter observation indicates the high propensity of the compounds to interact with water molecules from the solvents or the atmosphere as the evaporation experiments were performed at ambient conditions (30 to 40% RH) and no precautions were undertaken to evaporate at 0% RH (desiccant or dry nitrogen purge). Therefore, sublimation and dehydration experiments were attempted to produce unsolvated form(s) of **1** and **2**. For both compounds, sublimation experiments at temperatures ≥150 °C resulted in an anhydrous form (**1-I** and **2-I**). Furthermore, storing the hydrate at driest conditions (0% RH, over P_2_O_5_) for weeks at RT or heating the hydrates above 120 °C resulted in these solvent free forms. However, it has to be noted that **1-I** and **2-I** are only stable at RT if the atmospheric moisture is very low. The latter information is crucial for handling the anhydrous forms of **1** and **2**.

### 2.1. Thermal Behavior of the Hydrates of o-Phenanthroline HCl and Neocuproine HCl

The temperature-dependent stability and transformation pathways of the hydrates **1-Hy1**, **2-Hy1** and **2-Hy3** were investigated using HSM, DSC and TGA.

#### 2.1.1. Hot-Stage Microscopy

**Figure 2:**
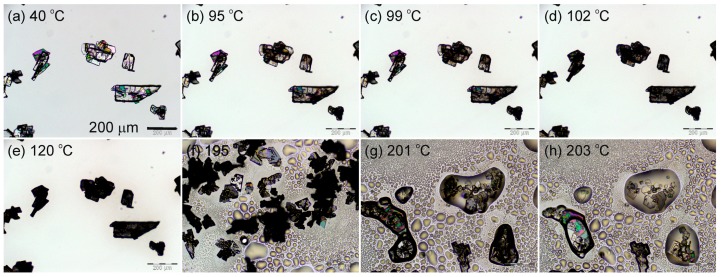
Microphotographs of **1-Hy1**. (**a**–**e**) Dehydration in the temperature range from 30 °C to 120 °C, (**f**–**h**) condensation, sublimation and melting of **1-I**.

Exemplarily, for **1-Hy1** and **2-Hy3** (**2-Hy1**), Figure 2 illustrates the optical dehydration behavior of **1-Hy1**. On heating, the platy hydrate crystals turn opaque, but maintain the outer shape of the original crystals, which is typical for the dehydration of stoichiometric hydrates and termed ‘pseudomorphosis’. The dehydration process starts at about 90 °C, with a maximum dehydration rate between 110 and 120 °C. The transformation is indicated by the appearance of dark spots on the surface of the hydrate crystals. Upon further heating, condensation and sublimation of **1-I** starts at 150 °C. At 200 °C decomposition and melting of the **1-I** crystals occurs. By heating hydrate crystals embedded in low viscous silicon oil (not shown), the formation of gas bubbles is observed at about 85 °C indicating the release of water from the hydrate. In high viscous silicon oil and fast heating rates the peritectic melting can be observed at 167 to 170 °C. 

The dehydration of **2-Hy3** starts already at 45 °C and a transformation to **2-Hy1** is observed, which then dehydrates to **2-I** at temperatures above 100 °C. Upon further heating, sublimation of fine **2-I** crystals can be observed. Bigger crystals of **2-I** melt above 190 °C and simultaneously thermal decomposition occurs. By embedding **2-Hy3** in high viscosity silicon oil, it could be confirmed that dehydration proceeds in two steps. At temperatures around 40 to 55 °C bubble formation occurs and upon further heating, the remaining hydrate water is released at temperatures around 110 °C, confirming that dehydration of **2-Hy3** proceeds via the lower hydrate **2-Hy1**.

#### 2.1.2. Differential Scanning Calorimetry and Thermogravimetric Analysis

To study the influence of the atmospheric conditions on the dehydration behavior and associated processes, different experimental conditions were applied in the DSC investigations by using perforated or hermetically sealed crucibles, or varying the heating rates (DSC and TGA).

**Figure 3:**
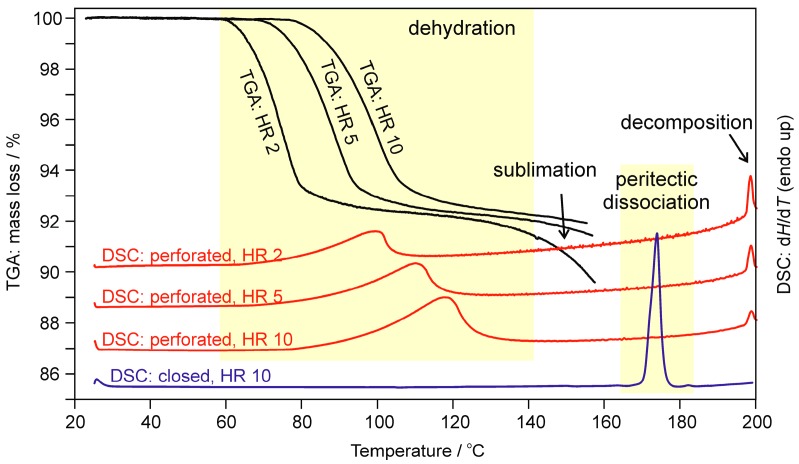
Differential Scanning Calorimetry (DSC) and Thermogravimetric Analysis (TGA) thermograms of **1-Hy1**. Numbers correspond to heating rates in °C min^−1^.

The TGA curves of **1-Hy1** (Figure 3) show a one-step dehydration process in the temperature range from 60 to 110 °C. The measured mass loss of one mole of water per mole **1-Hy1** confirms the presence of a monohydrate. Lowering the heating rate shifts the dehydration process to lower temperatures without effect on the mechanism of the reaction. Upon further heating, a second mass loss is observed, which corresponds to strong sublimation of **1-I**. The DSC curves recorded using perforated crucibles show a broad endothermic event in the temperature range 70 to 110 °C which coincides with the dehydration range seen in HSM and TGA investigations. At temperatures around 200 °C the sample starts to melt and decompose simultaneously. The peritectic dissociation peak of **1-Hy1** was recorded in closed DSC crucibles at 170.4 ± 0.5 °C (onset temperature).

**Figure 4:**
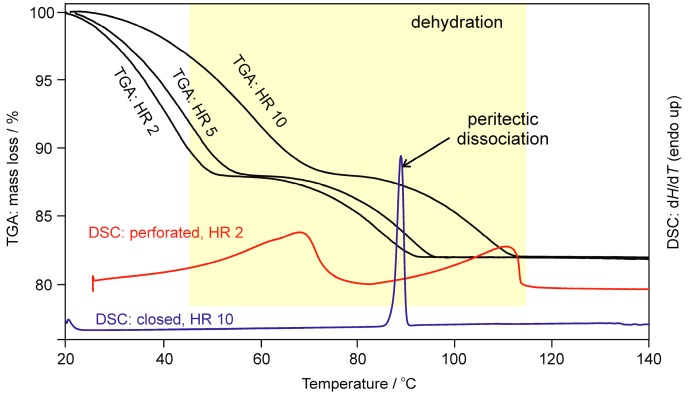
DSC and TGA thermograms of **2-Hy1**. Numbers correspond to heating rates in °C min^−1^.

**Figure 5:**
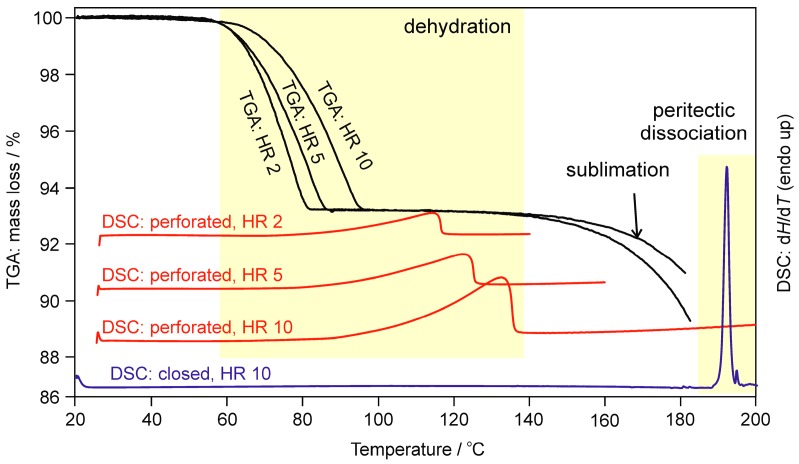
DSC and TGA thermograms of **2-Hy3**. Numbers correspond to heating rates in °C min^−1^.

The TGA curve of **2-Hy3** exhibits a two-step dehydration process (Figure 4). From the measured mass loss (first step two moles of water and second step one mole of water per mole **2**) it was clearly confirmed that the compound occurs as stoichiometric tri- and monohydrate. The TGA investigations also confirm the transformation steps from **2-Hy3** to **2-Hy1** and subsequently to **2-I**. Dehydration of **2-Hy3** starts immediately by exposing the hydrate to dry atmospheric conditions (N_2_ purge). By contrast, **2-Hy1** is stable up to about 60 °C under N_2_ purge (Figure 5). The DSC curve of **2-Hy1** (Figure 5) recorded in perforated crucibles, shows two endotherms in the temperature range 45 to 115 °C, corresponding to the two dehydration reactions. The peritectic dissociation of **2-Hy3** occurs already at 87.6 ± 0.1 °C, whereas **2-Hy1** dissociates at much higher temperatures (190.9 °C). 

The fact that the two monohydrates **1-Hy1** and **2-Hy1** show (referred to organic hydrates) rather high dehydration (90 °C and higher) and very high peritectic dissociation temperatures (170–190 °C) indicates that these hydrates are stable and the prevailing solid-state forms of the two compounds.

### 2.2. Moisture-Dependent Phase Transformations

#### 2.2.1. Gravimetric Moisture Sorption/Desorption Experiments

**Figure 6:**
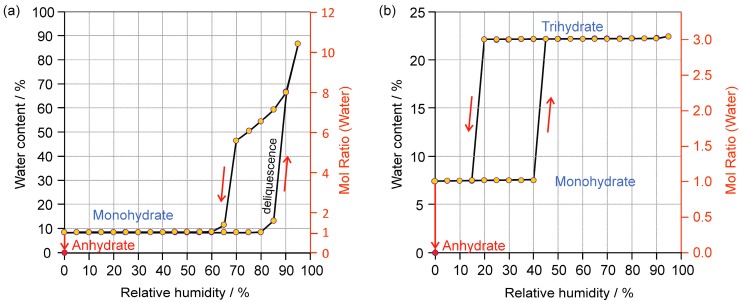
Gravimetric moisture sorption/desorption isotherms of (**a**) **1** and (**b**) **2**. Arrows indicate the course of the experiments. The anhydrates of the two compounds were obtained by storing the samples over P_2_O_5_.

The hydration and dehydration pathways of the two HCl salts were monitored as a function of RH. **1-Hy1** is stable over a wide RH range at 25 °C, i.e., no desorption is observed in the RH range 80% to 1% (Figure 6a). On increasing the RH above 80%, deliquescence of **1** occurs. This information is crucial for handling and storing **1**. At room temperature a dehydration of **1-Hy1** can only be observed at extremely low water vapor pressures over P_2_O_5_ where complete dehydration occurs within three weeks. The dehydration product corresponds to **1-I**. Upon exposure of **1-I** to moisture the back-transformation to **1-Hy1** occurs within less than 10 min at 20% RH and 25 °C.

At ambient conditions **2-Hy1** and **2-Hy3** can co-exist (Figure 6b). **2-Hy1** absorbs water at RH values above 40% forming **2-Hy3**. The back-transformation of **2-Hy3** to **2-Hy1** occurs at an RH <20%. Thus, the critical water activity lies in between 0.2 and 0.4. The hydration and dehydration kinetics between the two hydrates is relatively fast (<24 h). The hysteresis range between sorption and desorption lies within commonly observed humidity ranges, which explains why both forms can co-exist and are found in the commercial product. The discontinuous course of the isotherm and the hysteresis between the sorption and desorption curves clearly indicates the presence of stoichiometric hydrates [6]. The latter implies that a structural change occurs during the transformations and that the two hydrates are distinct phases. Similar to **1-Hy1**, it is possible to dehydrate **2-Hy1** to **2-I** at very low water vapor pressures (storage over P_2_O_5_) at 25 °C. The complete dehydration takes at least two weeks whereas the back-transformation of **2-I** to **2-Hy1** occurs within minutes if **2-I** is exposed to elevated moisture conditions.

#### 2.2.2. Moisture Controlled PXRD Experiments

**Figure 7:**
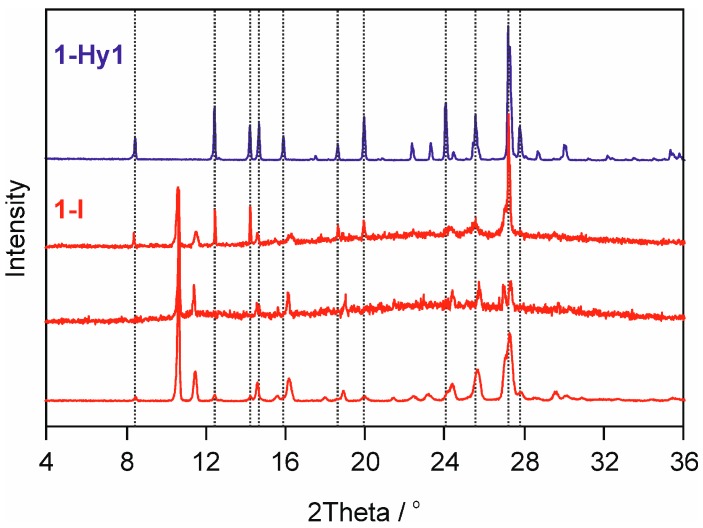
PXRD diffractograms of **1-Hy1** and **1-I**. Note that some of the anhydrate patterns contain significant amounts of **1-Hy1**. Dotted lines mark characteristic **1-Hy1** peak positions.

The gravimetric moisture sorption/desorption studies were complemented with PXRD measurements. The anhydrate samples were prepared via thermal dehydration, either in DSC crucibles or between two thin glass slides, and measured either directly in the aluminum DSC crucibles or the glass preparations. The PXRD data in Figure 7 clearly indicate the presence of two distinct phases, **1-I** (CUZDIK [10]) and **1-Hy1** (PHOLCL/PHOLCL01 [11,12]).

**Figure 8:**
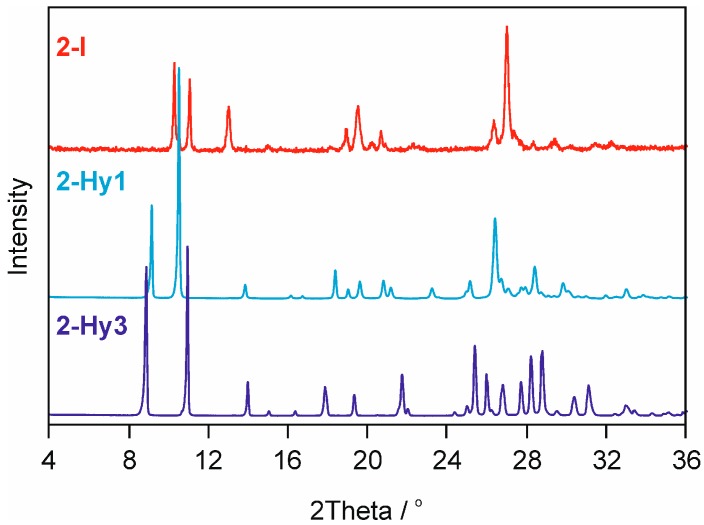
PXRD diffractograms of **2-I**, **2-Hy1** and **2-Hy3**.

The hydrate PXRD diffractograms (Figure 8) of **2** were recorded at 60% and 5% RH for **2-Hy3** and **2-Hy1**, respectively. In agreement with the thermal and gravimetric sorption/desorption data the two hydrate phases are distinguishable. Furthermore, **2-I** can be easily discriminated from the hydrate phases and based on the PXRD patterns no isostructurality between the solid forms of **1** and **2** can be expected.

### 2.3. Crystal Structures and Intermolecular Energy Calculations

Single-crystal structures have been published for **1**, but no structural information about **2** could be found in scientific literature prior to this study. The literature structures of **1-I** and **1-Hy1** are discussed together with the single-crystal structure of **2-Hy1** and a structure model for **2-I** derived from CSP.

#### 2.3.1. *o*-Phenanthroline HCl Crystal Structures

**Figure 9:**
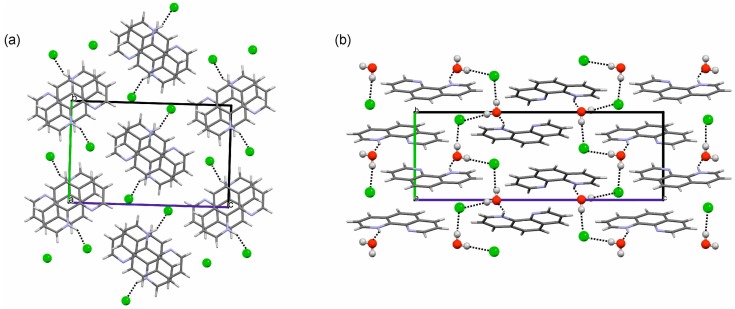
Packing diagrams of (**a**) **1-I** [10] viewed along *a* and (**b**) **1-Hy1** [11,12] viewed along *a*. Dotted lines indicate H-bonding interactions.

The anhydrate (**1-I**) crystallizes in the monoclinic space group *P*2_1_/*c*, with Z’ = 1 [10]. The cations, planar molecules, are arranged in stacks (Figure 9a). The Cl^−^ ion is coordinated by six phenanthrolinium cations. The strongest pairwise intermolecular interaction, accounting for −411.5 kJ mol^−1^, is the ionic N–H^+^···Cl^−^ hydrogen bonding interaction (Supplementary Material, Table S2).

Similar to **1-I**, the cations in **1-Hy1** (*P*2_1_/*c*, Z’ = 1) are arranged in stacks [11,12]. The Cl^−^ ion is surrounded by six cations, but in contrast to **1-I**, the H-bonding interaction in **1-Hy1** is not formed between the phenanthrolinium and Cl^−^, but instead the water molecule forms a N–H^+^···O H-bonding interaction (Figure 9b). The pairwise intermolecular interaction energy for the latter was estimated to be −50.5 kJ mol^−1^ (Supplementary Material, Table S2). Furthermore, the water molecule is strongly connected to the Cl^−^, two strong O–H···Cl^−^ H-bonding interactions (−72.3 kJ mol^−1^ and −70.4 kJ mol^−1^), forming zig-zag chains which propagate parallel to the *b* axis. Overall, the two **1** packings do not show structural resemblance, suggesting a destructive dehydration mechanism in agreement with the presence of a stoichiometric hydrate.

#### 2.3.2. Neocuproine HCl Monohydrate Crystal Structure

**Figure 10:**
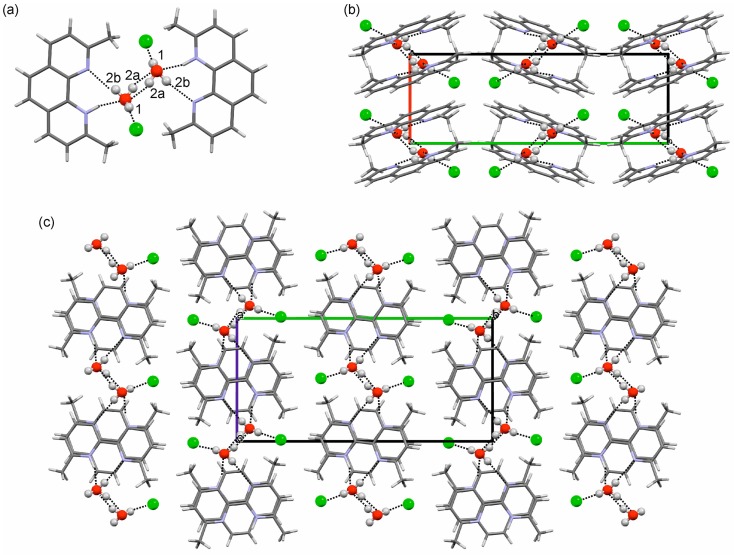
H-bonding and packing diagrams of **2-Hy1**: (**a**) Water proton disorder; (**b**,**c**) Packing diagrams in (**b**) viewed along the crystallographic *c* and in (**c**) along *a* axis. Dotted lines indicate H-bonding interactions.

The monohydrate of **2** crystallizes in the monoclinic space group *P*2_1_/*c* with each one cation, one Cl^−^ anion and one water molecule in the asymmetric unit, in agreement with a water to compound ratio of 1:1 as determined in the TGA experiments. As seen for **1,** the planar cation forms inversion related stacks of molecules in **2-Hy1** (Figure 10b,c) and the Cl^−^ ion is surrounded by seven cations. The water molecule acts as an acceptor to the N–H^+^ group of the cation. The pairwise interaction energy of the latter interaction, N–H^+^···O, was estimated to be −46.4 kJ mol^−1^ (Supplementary Material, Table S3). The strongest interaction involving water is formed between water and Cl^−^, O–H···Cl^−^ (−63.4 kJ mol^−1^). One H-atom of the water molecule is disordered over two positions, denoted 2a and 2b hereafter (Figure 10a). Orientation 2a connects adjacent water molecules through an O–H···O H-bond, with a pairwise interaction energy of −25.2 kJ mol^−1^. The second orientation, 2b, also forms a H-bonding interaction, O–H···N. If formed, the pairwise interaction energy was calculated to be −59.9 kJ mol^−1^. The fact that the latter energy is only diminished by −13.5 kJ mol^−1^ if orientation 2a is present (pairwise interaction energy between cation and water: −46.4 kJ mol^−1^ instead of −59.9 kJ mol^−1^) rationalizes why the water–water interaction is formed. The symmetry relation, inversion, between the two water molecules dictates that only every alternate 2a position can be occupied and, thus, resulting in the proton disorder. As expected, the strongest pairwise intermolecular interactions involve the two charged species (<−300 kJ mol^−1^, Supplementary Material, Table S3).

The two monohydrates, **1-Hy1** and **2-Hy2**, can both be classified as isolated-site hydrates [17]. Similar as seen for other HCl hydrates (e.g., morphinanes [16]), the addition of water to the crystal structure profoundly influences the H-bonding interaction preferentiality. The cation–anion H-bonding interactions, X–H^(+)^···Cl^−^, formed in the anhydrous forms, are replaced by water–cation and water–Cl^−^ interactions. The water molecules are located in between the charged species and, thus, are strongly held within the hydrate structures, rationalizing why dehydration occurs only at relatively high temperatures or at driest conditions at 25 °C.

#### 2.3.3. Computationally Generated Neocuproine HCl Anhydrate Structures

Due to the challenges encountered in handling **2-I**, we attempted to derive structural information by computationally generating hypothetical low-energy anhydrate structures, CSP. The crystal energy landscape (Table 1 and Supplementary Material, Figure S1) exhibits numerous thermodynamically feasible structures within the energy range expected for polymorphism [18]. Sixteen unique Z’ = 1 anhydrate packings were found within eight kJ mol^−1^ of the lowest-energy minimum structure. All low-energy structures feature (distinct) stacks of the planar neocuproinium cation and the Cl^−^ anion is in close proximity to the N–H^+^ group.

**Figure 11:**
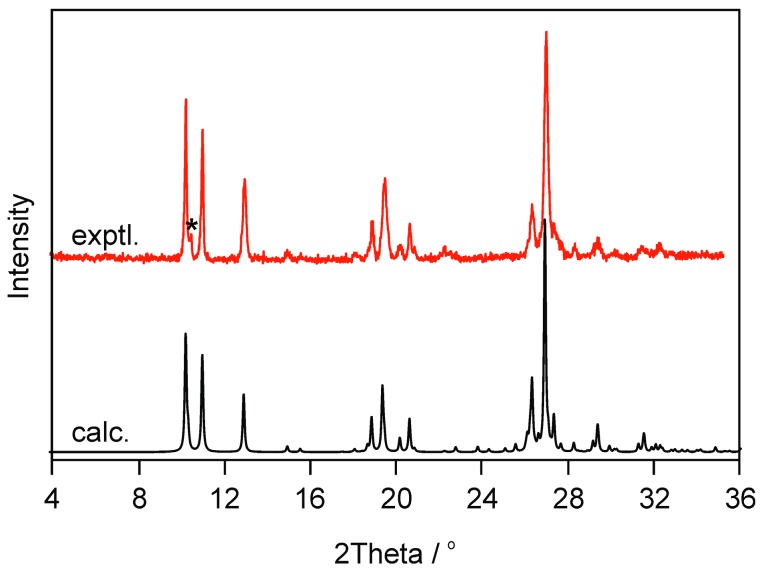
Experimental **2-I** PXRD pattern obtained at RT compared with the simulated RT pattern for the calculated structure 1_745. Asterisk ‘*’ indicates a **2-Hy1** phase impurity.

The experimental **2-I** PXRD diffractogram was successfully indexed: *P*$\overset{-}{1}$, a = 7.3060(32) Å, b = 9.4999(50) Å, c = 9.9049(41) Å, α = 113.241(19)°, β = 108.557(26)°, γ = 92.103(43)°, 23 °C. Based on the volume a Z’ = 1 structure can be assumed. The experimental PXRD pattern of **2-I** was compared to the patterns simulated from the computationally generated lowest-energy structures (Table 1). The best match, considering thermal effects, was found between **2-I** and the global minimum structure, 1_745 (Supplementary Material, Figure S2). The latter structure was re-optimized fixing the lattice parameters to the experimental RT values. The experimental and simulated powder patterns show a perfect match (Figure 11). To confirm that 1_745 is the experimental structure a rigid body Rietveld refinement was performed starting from the PBE-TS structure (see Section 2.2 of the Supplementary Material). The experimental and computed structures correspond to the same structure, with a rmsd_30_ [20] of 0.13 Å.

**Figure 12:**
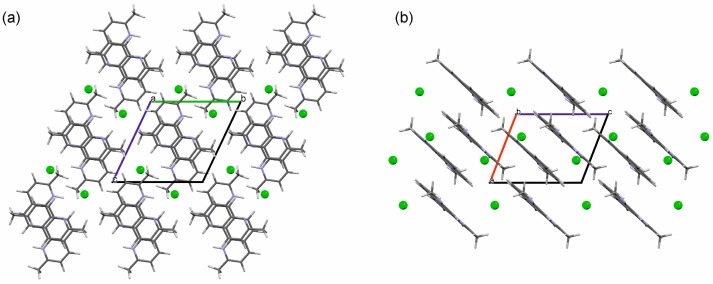
Packing diagrams of **2-I** viewed along (**a**) the *a* and (**b**) the *c* crystallographic axes.

Packing diagrams for **2-I** are shown in Figure 12. As already seen in **2-Hy1**, the Cl^−^ ion is coordinated by seven cations, with pairwise intermolecular energy contributions of <−200 kJ mol^−1^ due to the strong electrostatic contributions (Supplementary Material, Table S3). A common feature of the two anhydrates, **1-I** and **2-I**, is that the ionic (incl. H-bonding) interactions are solely responsible for the stability of the structures and overcome the strong repulsive interactions between the cations. Not even the π–π stacks, which provide strong pairwise dispersion contributions (*E*_D_, Supplementary Material, Tables S3 and S4), outmatch the repulsive electrostatic contributions between the cations. The introduction of water increases the coordination number of Cl^−^. The number of close contacts between the Cl^−^ ions and cations is the same for corresponding hydrate/anhydrate structures (six for **1** and seven for **2**), but in the case of **1-Hy1** and **2-Hy2** additional water–Cl^−^ H-bonding interactions are formed.

We did not succeed in producing **2-Hy3** single-crystals suitable for structure determination and did not attempt to compute the trihydrate crystal energy landscape (CSP) as this would imply computing structures with five distinct entities in the asymmetric unit.

### 2.4. Stability of Hydrates Estimated from Lattice Energy Calculations

Experimentally, is has been proven that **1-Hy1** and **2-Hy1** (**2-Hy3**) are very stable solid forms and that the neat forms of the two compounds exist only at high temperatures (above the peritectic dissociation temperatures of the hydrates) or at driest conditions. Computationally, we can estimate the potential energy differences (∆*U*) between crystal forms by comparing their lattice energies (*E*_latt_). The advantage of modeling is that we can generate structures that are experimentally not accessible. For **1** and **2** this means that the isostructural dehydrate structures were generated by removing the water from the monohydrates and optimizing the structures with space group constraints. A comparison of the isostructural dehydrates with the respective anhydrate structures reveals that the observed structures are more stable for both systems (**1**: −14.56 to −16.47 kJ mol^−1^ and **2**: −12.56 to −14.06 kJ mol^−1^, Table 2), implying that an isostructural dehydrate would be unstable and transform to the experimental structure, in agreement with the observed dehydration pathways.

Furthermore, it is possible to estimate the potential energy difference between hydrate and anhydrate forms based on their *E*_latt_ by comparing the lattice energy of the hydrate (*E*_latt_-Hy) to the lattice energies of the anhydrate (*E*_latt_-AH) and ice (*E*_latt_-ICE). If *E*_latt_-Hy < *E*_latt_-AH + *E*_latt_-ICE (assuming that hydrate formation is thermodynamically driven), then the hydrate is more stable than the anhydrate. Using the *E*_latt_ values given in Table 2 and a value of −59 kJ mol^−^^1^ [21,22] for ice (the used functional is known to overbind the ice crystal structures [23,24]) a potential energy difference (∆*U*) of −30 kJ mol^−^^1^ (**1**) and −20 kJ mol^−^^1^ (**2**) was calculated for the two hydrate/anhydrate systems. The ∆*U* values are in the range and even lower (more stable) than estimated for other stable organic hydrates (e.g., [16,25]). Thus, the lattice energy comparisons indicate the presence of stable hydrate structures.

Finally, the comparison between the hydrate and isostructural dehydrate (dehy) structures and ice allows us to estimate the intermolecular energy contributions of the water molecules to the hydrate structures. The obtained values, ca. −45 kJ mol^−1^ are very low (stable) for monohydrates, rationalizing the key role of water in the structures and the fast hydration kinetics.

## 3. Discussion

### 3.1. Hydrate Formation and Structural Role of Water in Phenanthrolinium Based HCl Salts 

The two model HCl salts form exceptionally stable hydrates. Often a mismatch in the number of H-bonding donor and acceptor groups is seen as a reason for hydrate formation [26]. In case of the two studied compounds it is not the mismatch but the close proximity of the protonated donor and acceptor groups and steric hindrance that prevent stronger attractive forces/H-bonding (electrostatics) between the phenanthrolinium or neocuproinium cations. Thus, the only non-repulsive interactions are formed between the Cl^−^ anion and the cation. A survey of the CSD [9] revealed that N–H^+^···N H-bonding is possible but requires the presence of an ionic co-crystal, i.e., phenanthrolinium cation and neutral phenanthroline as seen for example in following structures: BIBROT [27], IHUYIV [28], NIDXUT [14], QELBUF [15], etc. Exemplarily, for the two chemically related molecules the anhydrate crystal energy landscape was computed for **2** and the calculations confirm that the anion (Cl^−^) is exclusively located in close proximity to the N–H^+^ group and that the cations are always arranged in a stacked manner.

A statistical survey of all small pharmaceuticals officinal in the European Pharmacopeia revealed that nearly one third of all salts form hydrates and that for the subgroup of the HCl salts the incidence increases to nearly 40% [29]. In the case of the phenanthrolinium cation the occurrence of hydrates was found to be even higher. Almost 45% of the 154 unique structures (metal-organic structures excluded) present in the CSD are hydrates. The water molecule, small in size and providing H-bonding donor and acceptor groups, can “replace” the position of the Cl^−^ in the neat structures as confirmed by the CSD hydrates. In the hydrate structures the water molecule “links” the two charged species (e.g., Figure 9 and Figure 10) through strong H-bonding interactions. Thus, the molecular features of the phenanthrolinium and neocuproinium cations (rigid, planar and H-bonding and donor groups adjacent) and of the water molecule can be seen as the driving force for hydrate formation.

### 3.2. Stability and Handling of o-Phenanthroline HCl and Neocuproine HCl Solid-State Froms 

The study highlights that with the applied experimental techniques, thermal analysis and gravimetric moisture sorption/desorption experiments, the kinetic and thermodynamic stability of the two hydrate/anhydrate systems can be unraveled, and that the key variables temperature and RH for investigating, handling and storing hydrate forming systems are covered [30,31]. Having a molecular understanding of how water vapor is sorbed in hygroscopic materials and the risk that water poses to the physical and chemical stability of a fine chemical product is essential [32,33]. Careful evaluation of the stoichiometry, stability relationships, and transformation pathways are mandatory for developing robust manufacturing processes, handling and storing any fine chemical (product).

Surprisingly, no information about solid forms, apart from the single-crystal structure determinations, and stability thereof can be found for the two investigated HCl salts, despite the fact that both substances undergo changes if exposed to moisture. **1-Hy1** is an exceptionally stable monohydrate form, but if the RH increases 80% then deliquescence occurs. **2-Hy3**, on the other hand, does not show deliquescence upon increasing the RH, but **2** exhibits a phase transformation with a fast transition kinetics between a mono- and trihydrate in the RH range between 20% and 40%. Knowing which of the hydrates is present at any stage of production is important, in particular for weighing and dosing operations. Avoiding variations in the water content for **2** may be difficult under processing and storing conditions and thus requires special efforts to control the environmental conditions (moisture).

Water-free forms of the two compounds exist, albeit only at driest conditions at RT or at temperatures which are not suitable for storing or handling organic compounds. Our study was not addressed to screen for solid forms other than hydrates, but based on the performed experiments and calculations anhydrate polymorphism cannot be excluded, c.f. Table 1 which has numerous distinct **2** packings within less than eight kJ mol^−1^ of the experimental anhydrate. The route to alternative forms and its identification is for sure complicated by the strong tendency to form hydrates. Thus, alternative neat forms may only be of academic interest. Solvate formation has been confirmed for **1**. However, as seen for the anhydrates **1-I** and **2-I**, the chloroform solvate was found to be very unstable and to transform immediately to the hydrate at ambient conditions. A similar behavior may be expected for other potentially existing solvate forms.

### 3.3. Role of Computational Chemistry for Characterizing Organic Solid Forms

Lattice energy calculations allow us to estimate potential energy differences at 0 K but do not provide information about temperature- and moisture-induced effects. However, in the case of the two HCl salts he high energy difference of 20 kJ mol^−1^ and more between the anhydrate and monohydrate forms is a strong indication of stable hydrates relative to the neat forms.

Knowledge of the crystal structure provides an insight into the molecular interactions of a crystalline phase and often allows gaining an understanding of the observed transformation pathways. Sometimes it may not be possible to grow single-crystals and then other routes can be applied. A complementary approach is combining X-ray diffraction (or alternatively solid state NMR [34,35] or electron diffraction [36]) with crystal structure prediction, as demonstrated for the hard to handle anhydrate **2-I**. The fact that the closest matching structure was found as global minimum illustrates that the applied method, which has been successfully applied for neutral single- and multi-components [37], is transferable to the chosen HCl salts. 

The calculations of the pairwise intermolecular energy contributions to the HCl salts showed that the energy can be up to ±400 kJ mol^−1^ (negative if stabilizing and positive if destabilizing). Due to the fact that ion–ion interactions are of course long-range and as the CE-B3LYP energies generated in this study were only calculated in relative close proximity, we did not estimate the lattice energies based on the latter calculations. Lattice energy differences between pairs of polymorphs are typically small, in half of the cases of pairs of polymorphs the energy difference was calculated to be less than 2 kJ mol^−1^ [38], indicating the high accuracy needed if energy differences between solid forms are computed. Thus, the lattice energies given in this study were derived from electronic calculations on the crystal (PBE-TS and PBE-D2). The application of the two methods, CE-B3LYP and DFT-D, gives a unique complementary insight at a molecular level into the pairwise and crystal energies, allows the comparison between crystal forms and the identification of key packing fragments.

## 4. Materials and Methods

### 4.1. Materials and Preparation of Solid Forms

1,10-Phenanthroline hydrochloride monohydrate was purchased from Aldrich (lot # STBF6335V, purity ≥ 97%, Vienna, Austria) and recrystallized from an ethanol/2-propanol mixture for purification. The obtained form corresponded to **1-Hy1**. Form **1-I** was prepared by heating **1-Hy1** to 120 °C or storing the hydrate over P_2_O_5_ at 25 °C for 3 weeks.

Neocuproine HCl monohydrate was purchased from Sigma-Aldrich (lot # BCBN7538V, purity ≥ 99.0%, Vienna, Austria). The hydrates of **2** were prepared by storing the compound over saturated LiCl (11% RH) and NaCl (75% RH) salt solutions, resulting in **2-Hy1** and **2-Hy3**, respectively. The anhydrate **2-I** was prepared by heating either of the two hydrates to 120 °C or storing the hydrate over P_2_O_5_ at 25 °C for 3 weeks.

The organic solvents used were all of analytical grade and purchased from Aldrich (Vienna, Austria). 

### 4.2. Thermal Analysis

For HSM investigations a BH2 polarization microscope (Olympus, Vienna, Austria), equipped with a Kofler hot-stage (Reichert, Vienna, Austria), was used. Photographs were taken with an Olympus DP71 digital camera (Olympus, Vienna, Austria).

DSC thermograms were recorded on a DSC 7 or Diamond DSC (Perkin-Elmer, Norwalk, CT, USA) controlled by the Pyris 7.0 software. Using a UM3 ultramicrobalance (Mettler, Greifensee, Switzerland), samples of approximately 2–10 mg were weighed into open/sealed aluminum capsules. The samples were heated using rates in between 2 and 10 °C min^−1^, with dry nitrogen as the purge gas (purge: 20 mL min^−1^). The two instruments were calibrated for temperature with pure benzophenone (m.p. 48.0 °C) and caffeine (236.2 °C), and the energy calibration was performed with indium (m.p. 156.6 °C, heat of fusion 28.45 J g^−1^). The errors on the stated temperatures and enthalpy values were calculated at the 95% confidence intervals (CI) and are based on at least three measurements.

TGA was carried out with a TGA7 system (Perkin-Elmer, Norwalk, CT, USA) using the Pyris 2.0 software. Approximately 3–5 mg of sample was weighed into a platinum pan. Two-point calibration of the temperature was performed with ferromagnetic materials (Alumel and Ni, Curie-point standards, Perkin-Elmer, Norwalk, CT, USA). Heating rates in between 2 and 10 °C min^−1^ were applied and dry nitrogen was used as purge gas (sample purge: 20 mL min^−1^, balance purge: 40 mL min^−1^).

### 4.3. Gravimetric Moisture Sorption/Desorption Experiments

Moisture sorption and desorption studies were performed with the automatic multisample gravimetric moisture sorption analyzer SPS23-10µ (ProUmid, Ulm, Germany). The moisture sorption analyzer was calibrated with saturated salt solutions according to the supplier’s recommendations. Approximately 200–300 mg of sample was used for each analysis. The measurement cycles were started at 40% RH with an initial stepwise desorption (decreasing humidity) to 0%, followed by a sorption cycle (increasing humidity) to 95% RH and a final sorption step to 0% RH. RH changes were set to 5% for all sorption/desorption steps. The equilibria conditions for each step were set to a mass constancy of ±0.001% over 60 min and a maximum time limit of 48 h.

### 4.4. Powder and Single-Crystal X-Ray Diffraction

PXRD patterns were obtained using an X’Pert PRO diffractometer (PANalytical, Almelo, Netherlands) equipped with a θ/θ coupled goniometer in transmission geometry, programmable XYZ stage with well plate holder, Cu-Kα_1,2_ radiation source with a focusing mirror and a solid state PIXcel detector. The patterns were recorded at a tube voltage of 40 kV and tube current of 40 mA, applying a step size of 2θ = 0.013° with 40 s, 80 s or 200 s per step in the 2θ range between 2° and 40°. For non-ambient RH measurements, a VGI stage (VGI 2000M, Middlesex, UK) was used. The anhydrate samples were either measured in aluminum DSC crucibles or between two thin glass slides.

The diffraction pattern of **2-I** was indexed with DICVOL04 and the space group was determined based on a statistical assessment of systematic absences [39] as implemented in the DASH structure solution package [40]. Pawley fits [41] and Rietveld refinements [42] were performed with Topas Academic V5 [43]. The background was modelled with Chebyshev polynomials and the modified Thompson-Cox-Hastings pseudo-Voigt function was used for peak shape fitting.

SCXRD experiments. Single-crystals of **2-Hy1** were obtained from cooling crystallization experiments of **2** from a close to the boiling point saturated 1-propanol/toluene solution. The data set (Mo radiation; λ = 0.7107 Å) was collected on an Oxford Diffraction Gemini-R Ultra diffractometer operated by the CrysAlis software [44]. The structure was solved by direct methods (SIR2011) [45] and refined by full-matrix least squares on *F*^2^ using SHELXL2013 [46] and the program package WinGX [47]. All non-hydrogen atoms were refined anisotropically. N–H and O–H hydrogen atoms were located in difference maps and the water hydrogen atoms refined with distance restraints. All hydrogen atoms bound to carbon atoms were generated by a riding model in idealized geometries and their positions refined with *U*_iso_(H) = 1.5 *U*_eq_(C) for –CH_3_ groups and *U*_iso_(H) = 1.2 *U*_eq_(C) for aromatic H atoms. One of the water hydrogen atoms is disordered over two positions and was refined with an occupancy of 0.50:0.50. For details see Supplementary Material, Table S1. Furthermore, CCDC 1586332 contains the supplementary crystallographic data for this paper. These data can be obtained free of charge via http://www.ccdc.cam.ac.uk/conts/retrieving.html (or from the CCDC, 12 Union Road, Cambridge CB2 1EZ, UK; Fax: +44-1223-336033; E-mail: deposit@ccdc.cam.ac.uk). 

### 4.5. Computational Generation of the Neocuproine HCl Anhydrate Crystal Energy Landscape

Hypothetical crystal structures of **2** anhydrates, starting from the PBE0/6-31G(d,p) optimized molecular conformation, calculated using Gaussian09 [48], were generated with the program CrystalPredictor [49,50,51,52]. 300,000 structures were generated randomly in 48 space groups (*P*1, *P*$\overset{-}{1}$, *P*2_1_, *P*2_1_/*c*, *P*2_1_2_1_2, *P*2_1_2_1_2_1_, *Pna*2_1_, *Pca*2_1_, *Pbca*, *Pbcn*, *C*2/*c*, *Cc*, *C*2, *Pc*, *Cm*, *P*2_1_/*m*, *C*2/*m*, *P*2/*c*, *C*222_1_, *Pmn*2_1_*, Fdd*2, *Pnna*, *Pccn*, *Pbcm*, *Pnnm*, *Pmmn*, *Pnma*, *P*4_1_, *P*4_3_, *I*$\overset{-}{4}$, *P*4/*n*, *P*4_2_/*n*, *I*4/*m*, *I*4_1_/*a*, *P*4_1_2_1_2, *P*4_3_2_1_2, *P*3_1_, *P*3_2_, *R*3, *P*$\overset{-}{3}$, *R*$\overset{-}{3}$, *P*3_1_21, *P*322_1_, *R*3c, *R*$\overset{-}{3}$*c*, *P*6_1_, *P*6_3_, *P*6_3_/*m*), keeping the molecular geometry rigid. The structures were relaxed to a local minimum in the intermolecular lattice energy, calculated from the FIT [53] exp-6 repulsion-dispersion potential and atomic charges which had been fitted to the electrostatic potential around the PBE0/6-31G(d,p) charge density using the CHELPG scheme [54]. The energies of all structures within 20.0 kJ mol^−1^ of the global lattice energy minimum were refined (6343 structures), using DMACRYS [55] with a more realistic, distributed multipole model [56] for the electrostatic forces which had been derived using GDMA2 [57] to analyze the PBE0/6-31G(d,p) charge density. The most stable anhydrates (91 structures, 15 kJ mol^−1^ with respect to the global minimum) were optimized with periodic density functional calculations (CASTEP [58]). The Perdew–Burke–Ernzerhof (PBE) generalized gradient approximation (GGA) exchange-correlation density functional [59] and ultrasoft pseudopotentials [60], with the addition of the Tkatchenko and Scheffler (TS) [61] semi-empirical dispersion correction, were applied. The optimizations were considered complete when energies were converged to better than 2 × 10^–5^ eV per atom, atomic displacements converged to 1 × 10^–3^ Å, maximum forces to 5 × 10^–2^ eV Å^−1^, and maximum stresses were converged to 1 × 10^−1^ GPa. Isolated molecule minimizations to compute the isolated *o*-phenanthrolinium, neocuproinium, Cl^−^ and water (*U*_gas_) were performed by placing a single molecule/ion in a fixed cubic 35 × 35 × 35 Å^3^ unit cell, then optimized with the same settings as used for the crystal calculations.

Additional single point energy calculations were performed without optimization of the PBE-TS structures, with the number of k-points chosen to provide a maximum spacing of 0.07 Å^−1^ and a basis set cut-off of 780 eV, using the D2 dispersion correction [62].

### 4.6. Modeling of the Pairwise Intermolecular Interactions

The pairwise energy contributions to **1-I**, **1-Hy1**, **2-I** and **2-Hy1** were calculated using CrystalExplorer V17 [63,64,65]. The optimized atomic positions (PBE-TS) have been used in the intermolecular interaction energy calculations. The model energies have been calculated between all unique nearest neighbor molecular/ion pairs. The used model (CE-B3LYP) uses B3LYP/6-31G(d,p) molecular wave functions calculated by applying the molecular geometries extracted from the crystal structures. This approach uses electron densities of unperturbed monomers to obtain four separate energy components: electrostatic (E_E_), polarization (E_P_), dispersion (E_D_), and exchange-repulsion (E_R_). Each energy term was scaled independently to fit a large training set of B3LYP-D2/6-31G(d,p) counterpoise-corrected energies from both organic and inorganic crystals. 

## 5. Conclusions

The two chemically related HCl salts have a high affinity towards water, in other words under conditions of RH typically found during production and storage the hydrates are the stable forms. The characterization of the anhydrates (**1-I** and **2-I**) was complicated by the difficulty in handling the water-free forms, which immediately transform to hydrates if exposed to common moisture conditions. Lattice energy and pairwise intermolecular energy calculations on the monohydrates and the anhydrates rationalized the destructive dehydration mechanism and the high stability of the hydrates. The water molecules are integral to the stability of the hydrate structure.

This study is another demonstration of CSP-aided structure solution from PXRD data. In the case of **1-I** the quality of the PXRD data did not allow us to solve the structure from PXRD but by comparing the experimental data with the simulated PXRD patterns of the set of lowest-energy computed structures it was possible to identify the structure and thus **1-I** is characterized at an atomistic level. The fact that the experimental structure was found as lowest-energy structure in the lattice energy landscape affirms that the used method is applicable for HCl salts, which represent a challenge as strong electrostatic and other weak (e.g., dispersion) interactions have to be modeled accurately.

The two hydrates of neocuproine HCl are a nice demonstration that knowledge about hydration and dehydration conditions are crucial as phase transformations may occur at ambient (production and storage) conditions. Prior to our study, the higher hydrate **2-Hy3** has not been described in scientific literature at all, even though the reversible hydration/dehydration reaction occurs in the RH range of 20% to 40%. To control the phase (either **2-Hy1** or **2-Hy3**) special efforts are required and the latter is only possible if the environmental conditions (moisture or water activity) are controlled, otherwise variation in the water content, i.e., hydrate stoichiometry, cannot be avoided. 

To conclude, only the combination of a variety of experimental techniques, covering temperature- and moisture-dependent stability, and computational modeling allowed us to generate sufficient kinetic, thermodynamic and structural information to understand the principles of hydrate formation of the model HCl salts.

## Figures and Tables

**Table 1:** Computationally generated low-energy PBE-TS neocuproine HCl anhydrate structures.

ID ^a^	Space Group	a (Å)	b (Å)	c (Å)	𝛂 (°)	𝛃 (°)	𝛄 (°)	*E*_latt_ (kJ mol^−1^)	∆*E*_latt_ (kJ mol^−1^)	PI ^b^ (%)
1_745	*P* $\overset{-}{1}$	7.119	9.504	9.870	113.68	108.59	92.14	−538.7	0.00	76.5
2_106	*P*2_1_/*n*	9.474	7.345	16.370	90	92.53	90	−537.1	1.63	76.4
3_3345	*P* $\overset{-}{1}$	7.009	9.543	10.217	65.64	75.28	68.69	−536.9	1.81	75.4
4_3308	*P*2_1_/*n*	10.750	6.671	16.143	90	94.42	90	−535.3	3.44	75.3
5_4999	*P* $\overset{-}{1}$	6.945	9.461	10.265	114.74	103.22	98.47	−534.7	4.04	75.8
6_4078	*C*2/*c*	18.783	9.468	13.392	90	104.40	90	−533.3	5.39	75.4
7_343	*C*2/*c*	16.778	10.571	13.379	90	102.80	90	−533.2	5.50	75.3
8_399	*P* $\overset{-}{1}$	7.185	9.520	10.059	113.62	92.29	110.70	−532.8	5.89	75.3
9_5489	*P*2_1_/*n*	6.570	18.550	9.580	90	97.76	90	−532.7	6.02	75.4
10_268	*P*2_1_/*n*	6.813	10.484	16.581	90	100.95	90	−532.6	6.17	74.7
11_395	*I*2/*a*	13.158	10.074	17.514	90	94.58	90	−532.3	6.44	75.3
12_790	*P*2_1_2_1_2_1_	6.637	10.073	17.329	90	90.00	90	−531.9	6.83	75.1
13_212	*C*2/*c*	16.705	10.680	13.247	90	101.63	90	−531.8	6.90	75.3
14_139	*P*2_1_/*n*	7.256	16.884	9.469	90	94.08	90	−531.7	7.00	75.2
15_136	*P*2_1_/*n*	10.027	6.656	17.567	90	95.77	90	−531.3	7.46	74.3
16_2716	*Pna*2_1_	6.627	16.767	10.463	90	90	90	−531.2	7.54	74.8

^a^ Structure ID: rank PBE-TS _ rank Crystal Predictor; ^b^ Packing Index calculated using PLATON [19].

**Table 2:** Lattice energy calculations (*E*_latt_) and potential energy differences (∆*U*) between experimental and computed **1** and **2** crystal structures.

Structure	*E*_latt_/kJ mol^−1^		∆*U*_trs_ (Hy1→I) ^a^/kJ mol^−1^		∆*U*_trs_ (Hy1→dehy) ^a^/kJ mol^−1^		∆*U*_trs_ (dehy→1) ^b^/kJ mol^−1^	
	PBE-TS ^c^	PBE-D2 ^d^	PBE-TS ^c^	PBE-D2 ^d^	PBE-TS ^c^	PBE-D2 ^d^	PBE-TS ^c^	PBE-D2 ^d^
**1-I**	−509.67	−480.91	−29.28	−29.05	−	−	16.47	14.56
**1-dehy**	−493.20	−466.35	−	−	−45.75	−43.61	−	−
**1-Hy1**	−597.95	−568.96	−	−	−	−	−	−
**2-I**	−538.72	−529.93	−19.98	−20.79	−	−	14.06	12.56
**2-dehy**	−524.66	−517.37	−	−	−45.75	−43.61	−	−
**2-Hy1**	−617.70	−609.72	−	−	−	−	−	−

^a^ ∆*U*_trs_ = *E*_latt_-Hy − (*E*_latt_-AH or dehy − *E*_latt_-ICE); ^b^ ∆*U*_trs_ = *E*_latt_-dehy − *E*_latt_-AH; ^c^ lattice parameters and atomic positions optimized; ^d^ lattice parameters fixed to the PBE-TS lattice parameters and atomic positions optimized.

## Supplementary Materials

The supplementary materials are available online.

## Abbreviations

The following abbreviations are used in this manuscript:

*a*_w_	water activity
CE-B3LYP	CrystalExplorer B3LYP calculations
CSD	Cambridge Structural Database
CSP	crystal structure prediction
Dehy	isostructural dehydrate
DFT-D (PBE-TS/D2)	dispersion corrected density functional calculations (Perdew-Burke-Ernzerhof, Tkatchenko and Scheffler/Grimme D2 dispersion correction)
DSC	differential scanning calorimetry
*E*_latt_	lattice energy
HCl	hydrochloride
HSM	hot-stage microscopy
P_2_O_5_	phosphorus pentoxide
PI	packing index
PXRD	powder X-ray diffraction
RH	relative humidity
RT	room temperature
SCXRD	single-crystal X-ray diffraction
TGA	thermogravimetric analysis
∆*U*	potential energy difference
**1**	*o*-phenanthroline hydrochloride
**2**	neocuproine hydrochloride
**I**	anhydrate
**Hy1**	monohydrate
**Hy3**	trihydrate
