# Supplementary material for: New Insights into Solid Form Stability and Hydrate Formation: o-Phenanthroline HCl and Neocuproine HCl
Source: Molecules. 2017 Dec 15;22(12):2238. doi: 10.3390/molecules22122238 (PMC6149885; doi:10.3390/molecules22122238)
Supplement: Supplementary file 1 [file molecules-22-02238-s001.zip › Supplementary/Braun_Phen_Neo_HCl_Supplementary Material_revised.docx]

New Insights into Solid Form Stability and Hydrate Formation: *o*-Phenanthroline HCl and Neocuproine HCl

Doris E. Braun*, Katharina Raabe, Anna Schneeberger,Volker Kahlenberg and Ulrich J. Griesser

*** Correspondence:** doris.braun@uibk.ac.at

Table of Contents

[1. Neocuproine HCl Monohydrate Single Crystal Structure 2](#_Toc491413371)

[2. Neocuproine HCl Anhydrate Crystal Energy Landscape 3](#_Toc491413372)

[3. Computationally Generated Neocuproine HCl Anhydrate Structures 5](#_Toc491413373)

[4. Pair-wise Intermolecular Energy Calculations 7](#_Toc491413374)

# Neocuproine HCl Monohydrate Single Crystal Structure

**Table S1**: Crystallographic data for **2-Hy1**.

| Crystal data | |
| --- | --- |
| Chemical formula | C14H13N2·Cl·H2O |
| *M*r | 262.73 |
| Crystal system, space group | Monoclinic, *P*21/*c* |
| Temperature (K) | 173 (2) |
| *a*, *b*, *c* (Å) | 7.1090 (10), 19.2572 (19), 9.8947 (12) |
| β (°) | 110.513 (15) |
| *V* (Å3) | 1268.7 (3) |
| *Z* | 4 |
| Radiation type | Mo *K*α |
| Crystal size (mm) | 0.33 × 0.04 × 0.04 |
| Data collection | |
| Diffractometer | Xcalibur, Ruby, Gemini ultra |
| Absorption correction | Multi-scan  *CrysAlis PRO* 1.171.38.43f (Rigaku Oxford Diffraction, 2015) Empirical absorption correction using spherical harmonics, implemented in SCALE3 ABSPACK scaling algorithm. |
| *T*min, *T*max | 0.966, 1.000 |
| No. of measured, independent and  observed [*I* > 2σ(*I*)] reflections | 8683, 2800, 2064 |
| *R*int | 0.040 |
| (sin /λ)max (Å-1) | 0.687 |
| Refinement | |
| *R*[*F*2 > 2σ(*F*2)], *wR*(*F*2), *S* | 0.048, 0.116, 1.06 |
| No. of reflections | 2800 |
| No. of parameters | 179 |
| No. of restraints | 5 |
| H-atom treatment | H atoms treated by a mixture of independent and constrained refinement |
| max, min (e Å-3) | 0.30, -0.22 |

Computer programs: *SHELXL2013* (Sheldrick, 2013), *ORTEP* for Windows (Farrugia, 2012), *WinGX* publication routines (Farrugia, 2012).

# Neocuproine HCl Anhydrate

# Crystal Energy Landscape


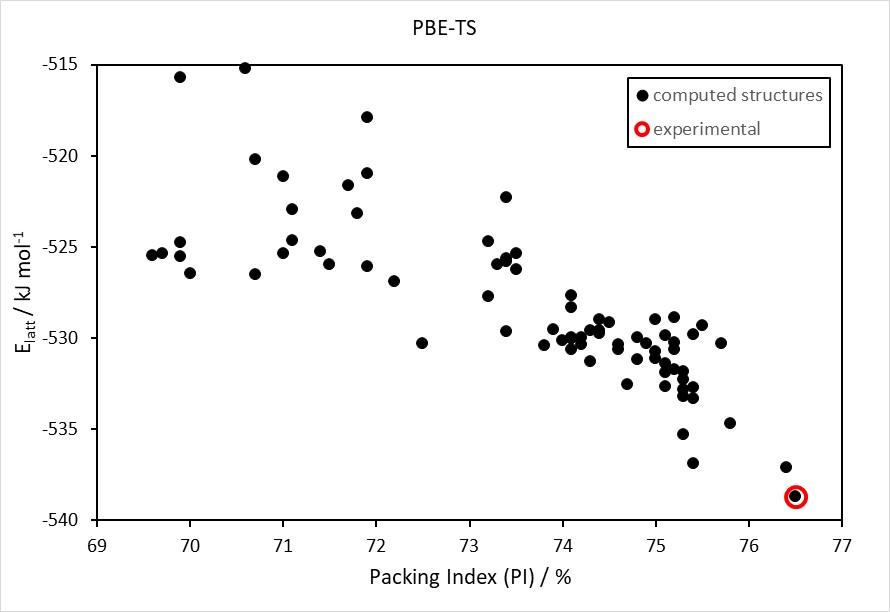


**Figure S1.** Anhydrate crystal energy landscape for neocuproine HCl. Each point corresponds to a hypothetical anhydrate structure. Experimental **2-I** structure encircled in red.


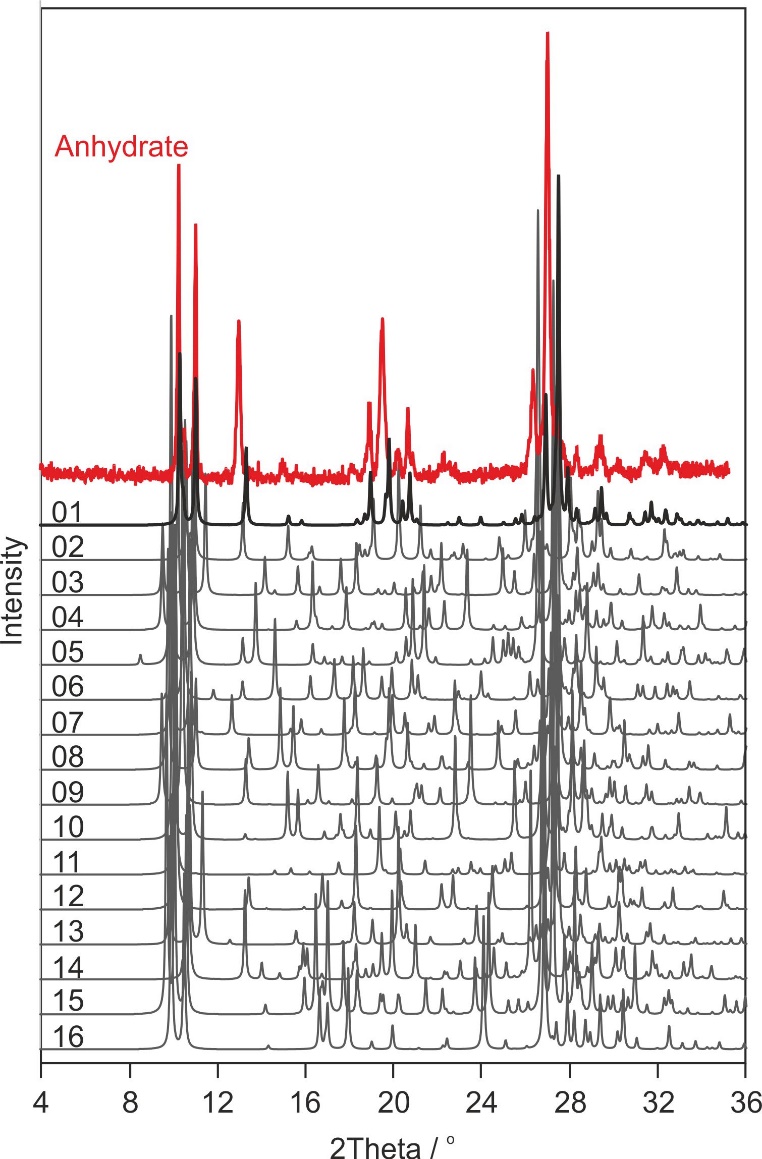


**Figure S2.** Experimental **2-I** PXRD pattern obtained at RT compared with the simulated 0 K patterns for the calculated lowest-energy anhydrate structures (Figure S1). Numbers correspond to rank.

# Rietveld Refinement

Pawley fits1 and Rietveld refinements2 were performed with Topas Academic V53. The background was modelled with Chebyshev polynomials and the modified Thompson-Cox-Hastings pseudo-Voigt function was used for peak shape fitting. For the Rietveld refinements the neocuproinium molecule and chloride ion were treated as rigid body molecules using the PBE-TS optimized conformations of the **2-I** structure. The isotropic temperature factor (*B*iso) for was set to 3.25 for all atoms. The final refinement included a total of 46 parameters (26 profile, 6 cell, 1 scale, 1 isotropic temperature factor, 6 position and 6 rotation). The refinement converged at *R*wp = 19.70%, *R*exp = 19.26%, *R*p = 14.63% and gof = 1.02.

**Figure S3.** Observed (black points), calculated (red line) and difference (green) profiles for the Rietveld refinement of **2-I**.


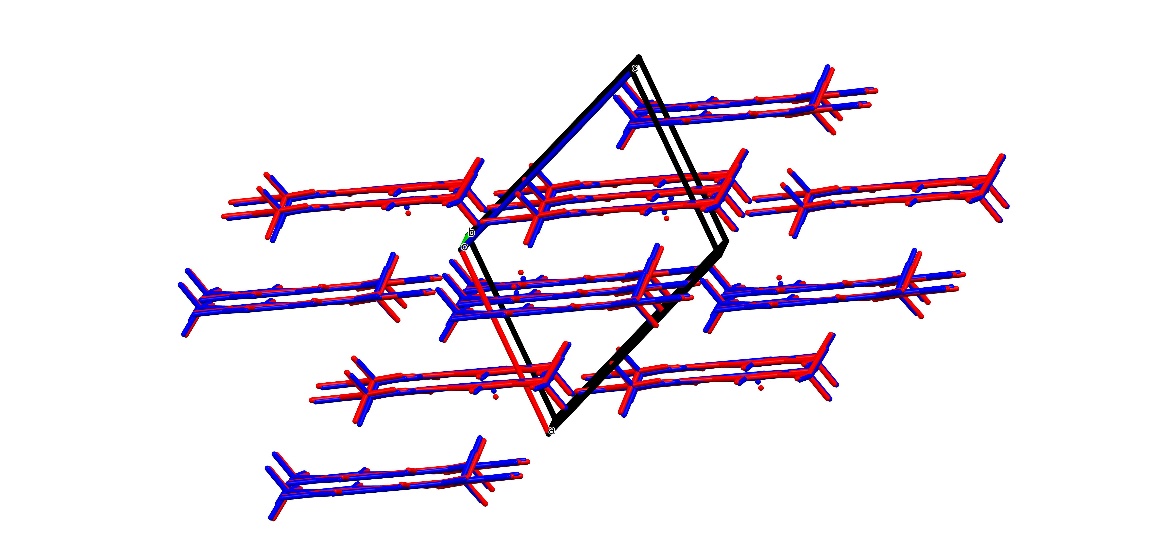


**Figure S3.** Overlay of the 30 molecule cluster of 2-I (Rietveld refinement, red) and PBE-TS structure (blue), rmsd30=0.13 Å.

# Computationally Generated Neocuproine HCl Anhydrate Structures

- 1. **Anhydrate 2-I: PBE-TS room temperature**

TITL 2-I_RT

CELL 1.54180 7.3060 9.4999 9.9049 113.241 108.557 92.103

ZERR 2 0.0000 0.0000 0.0000 0.000 0.000 0.000

LATT 1

SFAC C H N Cl

C1 1 0.32377 0.30939 0.03119 11.00000 0.0500

C2 1 0.29901 0.15303 0.00738 11.00000 0.0500

C3 1 0.39687 0.11062 0.12460 11.00000 0.0500

C4 1 0.53081 0.22420 0.26984 11.00000 0.0500

C5 1 0.63288 0.18734 0.39656 11.00000 0.0500

C6 1 0.75898 0.30067 0.53620 11.00000 0.0500

C7 1 0.79330 0.45774 0.55729 11.00000 0.0500

C8 1 0.92203 0.57986 0.69881 11.00000 0.0500

C9 1 0.94419 0.72886 0.71049 11.00000 0.0500

C10 1 0.83969 0.75987 0.58080 11.00000 0.0500

C11 1 0.69379 0.49936 0.43394 11.00000 0.0500

C12 1 0.55870 0.37981 0.28982 11.00000 0.0500

C13 1 0.21082 0.35890 -0.08982 11.00000 0.0500

C14 1 0.85904 0.92062 0.59032 11.00000 0.0500

H1 2 0.46199 0.53443 0.19379 11.00000 -1.20000

H2 2 0.19761 0.06737 -0.10626 11.00000 -1.20000

H3 2 0.37243 -0.01026 0.10932 11.00000 -1.20000

H4 2 0.60134 0.06716 0.37937 11.00000 -1.20000

H5 2 0.82899 0.27305 0.63512 11.00000 -1.20000

H6 2 1.00241 0.55665 0.79884 11.00000 -1.20000

H7 2 1.04246 0.82309 0.82024 11.00000 -1.20000

H8 2 0.24426 0.48595 -0.04675 11.00000 -1.50000

H9 2 0.05246 0.32236 -0.11959 11.00000 -1.50000

H10 2 0.24534 0.30165 -0.19694 11.00000 -1.50000

H11 2 1.01330 0.97052 0.62292 11.00000 -1.50000

H12 2 0.80544 1.00043 0.67921 11.00000 -1.50000

H13 2 0.77275 0.91644 0.47555 11.00000 -1.50000

N1 3 0.45043 0.41636 0.17117 11.00000 0.0500

N2 3 0.71816 0.64621 0.44525 11.00000 0.0500

Cl1 4 0.32809 0.75433 0.15134 11.00000 0.0500

END

- 1. **Anhydrate 2-I: PBE-TS 0 K**

TITL 2-I_0K

CELL 1.54180 7.1188 9.5035 9.8702 113.676 108.585 92.136

ZERR 2 0.0000 0.0000 0.0000 0.000 0.000 0.000

LATT 1

SFAC C H N Cl

C1 1 0.68689 0.19418 0.97558 11.00000 0.0500

C2 1 0.61273 0.39329 0.88320 11.00000 0.0500

C3 1 0.71386 0.35093 1.00034 11.00000 0.0500

C4 1 0.47404 0.27924 0.73670 11.00000 0.0500

C5 1 0.37113 0.31541 0.60895 11.00000 0.0500

C6 1 0.24384 0.20084 0.46705 11.00000 0.0500

C7 1 0.20707 0.04350 0.44505 11.00000 0.0500

C8 1 0.07568 -0.07987 0.30192 11.00000 0.0500

C9 1 0.05289 -0.22910 0.28960 11.00000 0.0500

C10 1 0.15930 -0.25915 0.42033 11.00000 0.0500

C11 1 0.30687 0.00288 0.56970 11.00000 0.0500

C12 1 0.44454 0.12335 0.71571 11.00000 0.0500

C13 1 0.80403 0.14364 1.09526 11.00000 0.0500

C14 1 0.14157 -0.42010 0.41027 11.00000 0.0500

H1 2 0.54147 -0.03164 0.81138 11.00000 -1.20000

H2 2 0.63858 0.51429 0.89898 11.00000 -1.20000

H3 2 0.82017 0.43678 1.11436 11.00000 -1.20000

H4 2 0.40408 0.43598 0.62700 11.00000 -1.20000

H5 2 0.17446 0.22786 0.36720 11.00000 -1.20000

H6 2 -0.00619 -0.05764 0.20101 11.00000 -1.20000

H7 2 -0.04694 -0.32397 0.17819 11.00000 -1.20000

H8 2 0.76694 0.01609 1.04971 11.00000 -1.50000

H9 2 0.96666 0.17933 1.12360 11.00000 -1.50000

H10 2 0.77127 0.20062 1.20441 11.00000 -1.50000

H11 2 -0.01608 -0.47115 0.37842 11.00000 -1.50000

H12 2 0.19582 -0.50014 0.32013 11.00000 -1.50000

H13 2 0.23240 -0.41462 0.52558 11.00000 -1.50000

N1 3 0.55502 0.08689 0.83464 11.00000 0.0500

N2 3 0.28195 -0.14415 0.55787 11.00000 0.0500

Cl1 4 0.33649 0.25376 0.15158 11.00000 0.0500

END

# Pair-wise Intermolecular Energy Calculations

Table S2. Selected pair-wise intermolecular interaction energiesa of 1-I and 1-Hy1.

| **Inter-**  **action** | | **Molecules Involved** | | **Distance (Å)** | | ***E*E**  **(kJ mol–1)** | | ***E*P**  **(kJ mol–1)** | | ***E*D**  **(kJ mol–1)** | | ***E*R**  **(kJ mol–1)** | | ***E*totb**  **(kJ mol–1)** | |
| --- | --- | --- | --- | --- | --- | --- | --- | --- | --- | --- | --- | --- | --- | --- | --- |
| **Phenanthroline HCl Anhydrate (1-I)** | | | | | | | | | | | | | | |
| 1 | **phen+ ··· Cl–** | | **4.56** | | **–373.1** | | **–83** | | **–7.5** | | **82.5** | | **–411.5** | |
| 2 | phen+ ··· Cl– | | 6.33 | | –284.4 | | –41 | | –5.4 | | 17.6 | | –324.9 | |
| 3 | phen+ ··· Cl– | | 6.12 | | –285.6 | | –47.2 | | –4.7 | | 31.9 | | –321.3 | |
| 4 | phen+ ··· Cl– | | 5.84 | | –253.7 | | –44.5 | | –5.2 | | 23 | | –291.5 | |
| 5 | phen+ ··· Cl– | | 5.73 | | –225.1 | | –26.5 | | –2.8 | | 2 | | –258.8 | |
| 6 | phen+ ··· Cl– | | 6.41 | | –221.5 | | –33.1 | | –4.7 | | 8.2 | | –257.6 | |
|  |  ···  | | 3.49 | | –233.9 | | –36.9 | | –76.4 | | 57.4 | | 188.9 | |
|  |  ···  | | 3.44 | | 226.4 | | –31.7 | | –73.4 | | 53.4 | | 185.0 | |
| **Phenanthroline HCl Monohydrate (1-Hy1)** | | | | | | | | | | | | | | |
| 1 | phen+ ··· Cl– | | 4.38 | | –282.5 | | –48.1 | | –6.6 | | 10.4 | | –333.7 | |
| 2 | phen+ ··· Cl– | | 5.82 | | –279.6 | | –46.2 | | –5.6 | | 20.8 | | –321.8 | |
| 3 | phen+ ··· Cl– | | 6.34 | | –281.9 | | –34.7 | | –3.7 | | 14.2 | | –318.2 | |
| 4 | phen+ ··· Cl– | | 5.83 | | –260.6 | | –46.6 | | –5.8 | | 23.1 | | –300.8 | |
| 5 | phen+ ··· Cl– | | 6.63 | | –252.6 | | –31.2 | | –3.9 | | 5.9 | | –290 | |
| 6 | phen+ ··· Cl– | | 6.98 | | –201.9 | | –21.5 | | –2.4 | | 1.8 | | –230.3 | |
| 7 | water ··· Cl– | | 3.02 | | –81.1 | | –29.9 | | –2.2 | | 60.7 | | –72.3 | |
| 8 | water ··· Cl– | | 3.08 | | –73.9 | | –27.8 | | –2.2 | | 48.9 | | –70.4 | |
| 9 | water ··· phen+ | | 4.28 | | –59.7 | | –22.1 | | –10 | | 60.9 | | –50.5 | |
| 10 | water ··· phen+ | | 4.88 | | –11.5 | | –3.4 | | –6.1 | | 5.1 | | –16.8 | |
| 11 | water ··· phen+ | | 6.53 | | –12.1 | | –3.3 | | –5.6 | | 6.4 | | –16.2 | |
| 12 | water ··· phen+ | | 6.1 | | –0.1 | | –1.2 | | –1.9 | | 0.2 | | –2.5 | |
|  |  ···  | | 4.56 | | 206.2 | | –23.0 | | –51.8 | | 34.3 | | 177.2 | |

aElectrostatic (*E*E), polarization (*E*P), dispersion (*E*D) and exchange-repulsion energy (*E*R) contributions. b*E*tot = *k*E *E*E + *k*P *E*P + *k*D *E*D + *k*R *E*R, with *k* being scale factors.

Table S3. Selected pair-wise intermolecular interaction energiesa of 2-I and 2-Hy1.

| **Inter-**  **action** | **Molecules Involved** | **Distance (Å)** | ***E*E**  **(kJ mol–1)** | | ***E*P**  **(kJ mol–1)** | | ***E*D**  **(kJ mol–1)** | | ***E*R**  **(kJ mol–1)** | | ***E*totb**  **(kJ mol–1)** | |
| --- | --- | --- | --- | --- | --- | --- | --- | --- | --- | --- | --- | --- |
| **Neocuproine HCl Monohydrate (2-Hy1)** | | | | | | | | | | | |
| 1 | neoc+ ··· Cl– | 6.03 | –280 | –52.8 | | –6.1 | | 31.2 | | –321.1 | |
| 2 | neoc+ ··· Cl– | 6.26 | –274 | –51 | | –6.9 | | 23.5 | | –319 | |
| 3 | neoc+ ··· Cl– | 5.66 | –262.6 | –38.7 | | –4.3 | | 13.3 | | –301.8 | |
| 4 | neoc+ ··· Cl– | 5.93 | –256.1 | –51.6 | | –6.5 | | 28.8 | | –296.9 | |
| 5 | neoc+ ··· Cl– | 7.21 | –212.3 | –31.8 | | –3.9 | | 9.6 | | –245.4 | |
| 6 | neoc+ ··· Cl– | 6.12 | –207.1 | –27.1 | | –3.2 | | 5.5 | | –238.4 | |
| 7 | neoc+ ··· Cl– | 7.25 | –209.5 | –16.4 | | –1.4 | | 0.7 | | –234.5 | |
| 8 | neoc+ ··· Cl– | 7.46 | –182.2 | –21.1 | | –2.4 | | 2.6 | | –208.7 | |
| 9a | water ··· Cl– | 3.08 | –68.1 | –27.4 | | –2 | | 49.6 | | –63.4 | |
| 9b | water ··· Cl– | 3.08 | –67.8 | –27.4 | | –2 | | 49.5 | | –63.1 | |
| 10a | water ··· neoc+ | 3.51 | –93.8 | –26.1 | | –16.5 | | 118.1 | | –59.9 | |
| 10b | water ··· neoc+ | 3.58 | –65.1 | –20.5 | | –12.8 | | 79 | | –46.4 | |
| 11 | water ··· water | 2.66 | –54.1 | –12.6 | | –4.2 | | 72.8 | | –25.2 | |
| 12a | water ··· neoc+ | 5.57 | –7.4 | –1.2 | | –1.9 | | 0.2 | | –10.3 | |
| 12b | water ··· neoc+ | 5.59 | –7.2 | –1.2 | | –2 | | 0.3 | | –10 | |
| 13a | water ··· neoc+ | 5.48 | –4.7 | –1.4 | | –3.1 | | 0.9 | | –8.1 | |
| 13b | water ··· neoc+ | 5.11 | –1.7 | –1.8 | | –4.9 | | 2.7 | | –5.8 | |
|  |  ···  | 3.52 | 211.5 | –31.7 | | –101.7 | | 84.5 | | 163.8 | |
|  |  ···  | 3.59 | 223.5 | –36.0 | | –83.6 | | 50.2 | | 167.9 | |
| **Neocuproine HCl Anhydrate (2-I)** | | | | | | | | | | | |
| 1 | neoc+ ··· Cl– | 4.39 | –331 | –82.2 | | –9.3 | | 53.4 | | –386 | |
| 2 | neoc+ ··· Cl– | 4.7 | –280.2 | –58.6 | | –8.4 | | 15.2 | | –337.6 | |
| 3 | neoc+ ··· Cl– | 6.06 | –278.2 | –51.5 | | –5.9 | | 29.7 | | –318.9 | |
| 4 | neoc+ ··· Cl– | 6.65 | –255.2 | –37.4 | | –4.2 | | 15 | | –291.8 | |
| 5 | neoc+ ··· Cl– | 5.81 | –231.2 | –38.1 | | –4.9 | | 11.4 | | –269.9 | |
| 6 | neoc+ ··· Cl– | 7.19 | –205.5 | –25.2 | | –2.8 | | 2.5 | | –236.8 | |
| 7 | neoc+ ··· Cl– | 7.15 | –198.5 | –28.2 | | –3.4 | | 10.8 | | –227.1 | |
|  |  ···  | 3.59 | 221.7 | –31.5 | | –91.0 | | 61.6 | | 169.8 | |

aElectrostatic (*E*E), polarization (*E*P), dispersion (*E*D) and exchange-repulsion energy (*E*R) contributions. b*E*tot = *k*E *E*E + *k*P *E*P + *k*D *E*D + *k*R *E*R, with *k* being scale factors.

**References**

1. Pawley, G. S., Unit-Cell Refinement from Powder Diffraction Scans. *Journal of Applied Crystallography* **1981,** *14* (DEC), 357-361.

2. Rietveld, H. M., A Profile Refinement Method for Nuclear and Magnetic Structures. *Journal of Applied Crystallography* **1969,** *2*, 65-71.

3. Coelho, A. A. *Topas Academic V5*, Coelho Software: Brisbane, 2012.
